# The role of PCSK9 in heart failure and other cardiovascular diseases—mechanisms of action beyond its effect on LDL cholesterol

**DOI:** 10.1007/s10741-024-10409-7

**Published:** 2024-06-18

**Authors:** Mieczysław Dutka, Karolina Zimmer, Michał Ćwiertnia, Tomasz Ilczak, Rafał Bobiński

**Affiliations:** 1 Department of Biochemistry and Molecular Biology, Faculty of Health Sciences, University of Bielsko-Biala, Willowa St. 2, 43-309 Bielsko-Biała, Poland; 2 Department of Emergency Medicine, Faculty of Health Sciences, University of Bielsko-Biala, 43-309 Bielsko-Biała, Poland

**Keywords:** PCSK9, Atherosclerosis, Inflammation, Heart failure, PCSK9 inhibitors

## Abstract

Proprotein convertase subtilisin/kexin type-9 (PCSK9) is a protein that regulates low-density lipoprotein (LDL) cholesterol metabolism by binding to the hepatic LDL receptor (LDLR), ultimately leading to its lysosomal degradation and an increase in LDL cholesterol (LDLc) levels. Treatment strategies have been developed based on blocking PCSK9 with specific antibodies (alirocumab, evolocumab) and on blocking its production with small regulatory RNA (siRNA) (inclisiran). Clinical trials evaluating these drugs have confirmed their high efficacy in reducing serum LDLc levels and improving the prognosis in patients with atherosclerotic cardiovascular diseases. Most studies have focused on the action of PCSK9 on LDLRs and the subsequent increase in LDLc concentrations. Increasing evidence suggests that the adverse cardiovascular effects of PCSK9, particularly its atherosclerotic effects on the vascular wall, may also result from mechanisms independent of its effects on lipid metabolism. PCSK9 induces the expression of pro-inflammatory cytokines contributing to inflammation within the vascular wall and promotes apoptosis, pyroptosis, and ferroptosis of cardiomyocytes and is thus involved in the development and progression of heart failure. The elimination of PCSK9 may, therefore, not only be a treatment for hypercholesterolaemia but also for atherosclerosis and other cardiovascular diseases. The mechanisms of action of PCSK9 in the cardiovascular system are not yet fully understood. This article reviews the current understanding of the mechanisms of PCSK9 action in the cardiovascular system and its contribution to cardiovascular diseases. Knowledge of these mechanisms may contribute to the wider use of PCSK9 inhibitors in the treatment of cardiovascular diseases.

## Introduction

Proprotein convertase subtilisin/kexin type 9 (PCSK9) is a member of the proprotein convertase family [1, 2]. This family of proteins is responsible for the post-translational modification of various biologically active proteins, including the activation of inactive prohormones, such as proinsulin [3]. PCSK9 is involved in the regulation of low-density lipoprotein (LDL) cholesterol by binding to hepatic LDL receptor (LDLR), ultimately leading to its lysosomal degradation [1]. Gain-of-function (GOF) mutations in the PCSK9 gene have been identified as a genetic mechanism for familial hypercholesterolaemia. Many types of such mutations have been reported [4]. High levels of PCSK9 and the consequent enhanced degradation of LDLRs result in a significant increase in serum LDL cholesterol (LDLc), which is a potent atherogenic factor and contributes to atherosclerotic cardiovascular diseases such as coronary artery disease (CAD), cerebrovascular disease, and peripheral atherosclerosis [4, 5]. The importance of PCSK9 in the regulation of LDLc metabolism is also highlighted by some loss-of-function (LOF) mutations in the PCSK9 gene, which result in the loss of functional PCSK9 [6, 7]. An example is the monoallelic double variant R110C + V114A, which is associated with plasma LDLc concentrations of 14–16 ng/ml in individuals with such a PCSK9 variant confirmed [8–10]. There are other LOF mutations in the PCSK9 gene, such as ^R97/Y142X, C679X/C679X, rs11583680, rs11591147, rs2479409, and rs11206510, and these also result in very low plasma LDLc concentrations. People who lack functional PCSK9 as a result of such LOF mutations in the PCSK9 gene can lead normal lives, and these genetic variants protect them from developing cardiovascular diseases [2, 8]. There are also reports of an association between these PCSK9 genetic variants and the risk of developing metabolic disorders, t.2 diabetes, obesity, and heart failure with preserved ejection fraction (HFpEF) [11–15].

Researchers are interested in PCSK9 as a target for lipid-lowering therapy. This has led to the development of treatment strategies based on blocking PCSK9 with specific antibodies directed against this protein (alirocumab, evolocumab) and on blocking its production with small interfering RNAs (siRNAs) (inclisiran). Clinical trials evaluating these PCSK9-eliminating drugs have confirmed their high efficacy in reducing serum LDLc levels and improving the prognosis in patients with atherosclerotic cardiovascular diseases [2, 16–20]. Most studies have focused on the effect of PCSK9 on LDLRs and the resulting increase in serum LDLc concentrations. However, there is increasing evidence that at least some of the adverse cardiovascular effects of PCSK9 are due to mechanisms that are independent of the enhancement of LDLR degradation in hepatocytes and its effect on lipid metabolism [3]. Its direct atherosclerotic effect on the vessel wall is highlighted. PCSK9 has been shown to contribute to foam cell formation by increasing macrophage uptake of LDLc and inhibiting macrophage cholesterol efflux. In addition, PCSK9 induces the expression of pro-inflammatory cytokines and adhesion molecules that contribute to inflammation within the vessel wall. PCSK9 also induces apoptosis of endothelial cells (ECs) and stimulates differentiation of vascular smooth muscle cells (VSMCs) into a synthetic phenotype [3, 21]. Thus, these clinical benefits of PCSK9 elimination therapy (e.g. with anti-PCK9 antibodies) may also result from mechanisms independent of LDLc. Treatment based on PCSK9 elimination may therefore not only be a treatment for hypercholesterolaemia, but also a treatment for atherosclerosis and other cardiovascular diseases. The mechanisms of action of PCSK9 in the cardiovascular system are not fully understood. However, both LDLR-dependent and LDLR-independent actions of PCSK9 have been implicated in inducing hypercholesterolaemia, inflammation, EC and VSMC apoptosis, cardiomyocyte apoptosis, pyroptosis, and ferroptosis.

## The structure of PCSK9

The primary gene product for human PCSK9 is a protein, called pre-proPCSK9, which consists of 692 amino acids. It contains a signal peptide, prodomain, central catalytic domain, hinge region, and C-terminal cysteine- and histidine-rich domain (CHRD) [1, 2, 22]. After removal of the signal peptide, proPCSK9 is formed, which undergoes autocatalysis in the endoplasmic reticulum and is automatically cleaved at its internal site to produce a 15kD prodomain and mature PCSK9 of 60 kD. PCSK9 is the only representative of the PCSK family in which these two latter parts are always non-covalently attached to each other [1, 2, 23, 24]. Prodomain inhibits the catalytic activity of mature PCSK9 by closing the active centre of the catalytic domain. Human PCSK9 undergoes no further cleavage steps to release the prodomain and, therefore, does not become a fully active protease. Fundamental to the biological function of PCSK9 is its remaining in the prodomain-PCSK9 heterodimer form, which is proteolytically inactive. This means that PCSK9 acts as a protease only once, during the process of autocatalysis in the endoplasmic reticulum. This suggests that PCSK9 performs its LDLc-regulatory action through non-enzymatic mechanisms [2]. Attention is drawn to the N-terminal (Gly31-Thr60) segment of PCSK9, which can bind a variety of important ligands. PCSK9 loses a large fragment of this segment (aa33-53), consisting of 25% Glu and Asp residues, and therefore its affinity for LDLR increases sevenfold in the acidic environment of hepatocyte endosomes/lysosomes. This results in stronger LDLR binding and enhanced LDLR degradation [2, 25–27]. More than 40 GOF and LOF variants of PCSK9 are known [8, 28].

The CHRD domain consists of three tandem repeats that are tightly packed into three structurally similar modules (M1, M2, M3). Modules M1, M2, and M3 of CHRD show structural similarity to the homotrimer resistin, which is a cytokine associated with pro-inflammatory stimulation within atherosclerotic plaques [2, 21, 29–32].

## Tissue expression of PCSK9

The main source of circulating PCSK9 in the blood is the liver, but PCSK9 expression has also been confirmed in the kidneys, brain, small intestine, colon, ischaemic heart as well as in VSMCs, ECs, and macrophages [1, 33, 34]. Furthermore, ox-LDL induces PCSK9 expression in dendritic cells and dendritic cell maturation as well as T-cell activation [35]. It is thought that dendritic cells may be involved in local immune activation and atherosclerotic lesion formation in arteries. This occurs through the ox-LDL-induced pro-inflammatory activation of dendritic cells and T cells [35].

VSMCs are characterised by a significantly higher expression and secretion of PCSK9 than ECs. PCSK9 expression is inducible in both ECs and VSMCs. In ECs, it can be induced by such things as shear stress and oxidised LDL (oxLDL), which are known atherogenic factors. The importance of shear stress in the induction of PCSK9 secretion by VSMCs increases significantly when EC injury occurs, resulting in VSMCs being directly exposed to haemodynamic events related to blood flow [36]. An increase in PCSK9 expression in ECs is associated with their apoptosis and an increase in reactive oxygen species (ROS) production [36, 37]. Furthermore, an increase in PCSK9 expression in VSMCs leads to various pathological phenomena including the apoptosis, proliferation, and migration of VSMCs P-1, P-1(Ding2016)]. PCSK9 secreted by VSMCs reduces LDLR expression in macrophages. This PCSK9 expression in macrophages can also be induced by oxLDL as well as by various cytokines such as tumour necrosis factor-alpha (TNF-α), and this leads to increased expression of scavenger receptors (SRs) such as scavenger receptor class A (SR-A), CD36, and the lectin-like oxLDL receptor-1(LOX-1) which increase oxLDL uptake by macrophages [38]. As mentioned above, PCSK9 is also involved in oxLDL-induced maturation and activation of dendritic cells, which produce TNF-α, interleukin-1beta (IL-1β), and interleukin-6 (IL-6) when exposed to such activated dendritic cells. T-cells exposed to activated dendritic cells also produce interferon gamma (INF-ɣ) and interleukin-17 (IL-17) with polarisation into Th1 and Th17 subsets. The silencing of PCSK9 reverses all these pro-inflammatory effects of oxLDL in both dendritic cells and activated T-cells [35]. This highlights the important role of local PCSK9 expression and action in the vasculature, particularly as ECs, VSMCs as well as macrophages, and other immune cells are all involved in the development and progression of atherosclerosis and other vascular diseases. This indicates a direct involvement of PCSK9 in the pathogenesis of cardiovascular diseases.

## Selected target proteins for PCSK9

In addition to binding to hepatic LDLR and leading to its lysosomal degradation, PCSK9 also has the ability to bind to other proteins such as very low density lipoprotein receptor (VLDR) and apolipoprotein E receptor 2 (ApoER2), thereby enhancing their degradation [1]. Previous studies suggest that PCSK9 may also contribute to the degradation of other receptors such as beta-site amyloid precursor protein cleaving enzyme 1 (BACE1), CD81, CD36, amyloid precursor protein (APP), amyloid precursor-like protein 2 (APLP2), and epithelial sodium channel (ENaC) [1]. Up to this time, 15 proteins have been identified with which PCSK9 can interact [39–46].

## LDLR family as an example of a target protein for PCSK9

**Fig. 1:**
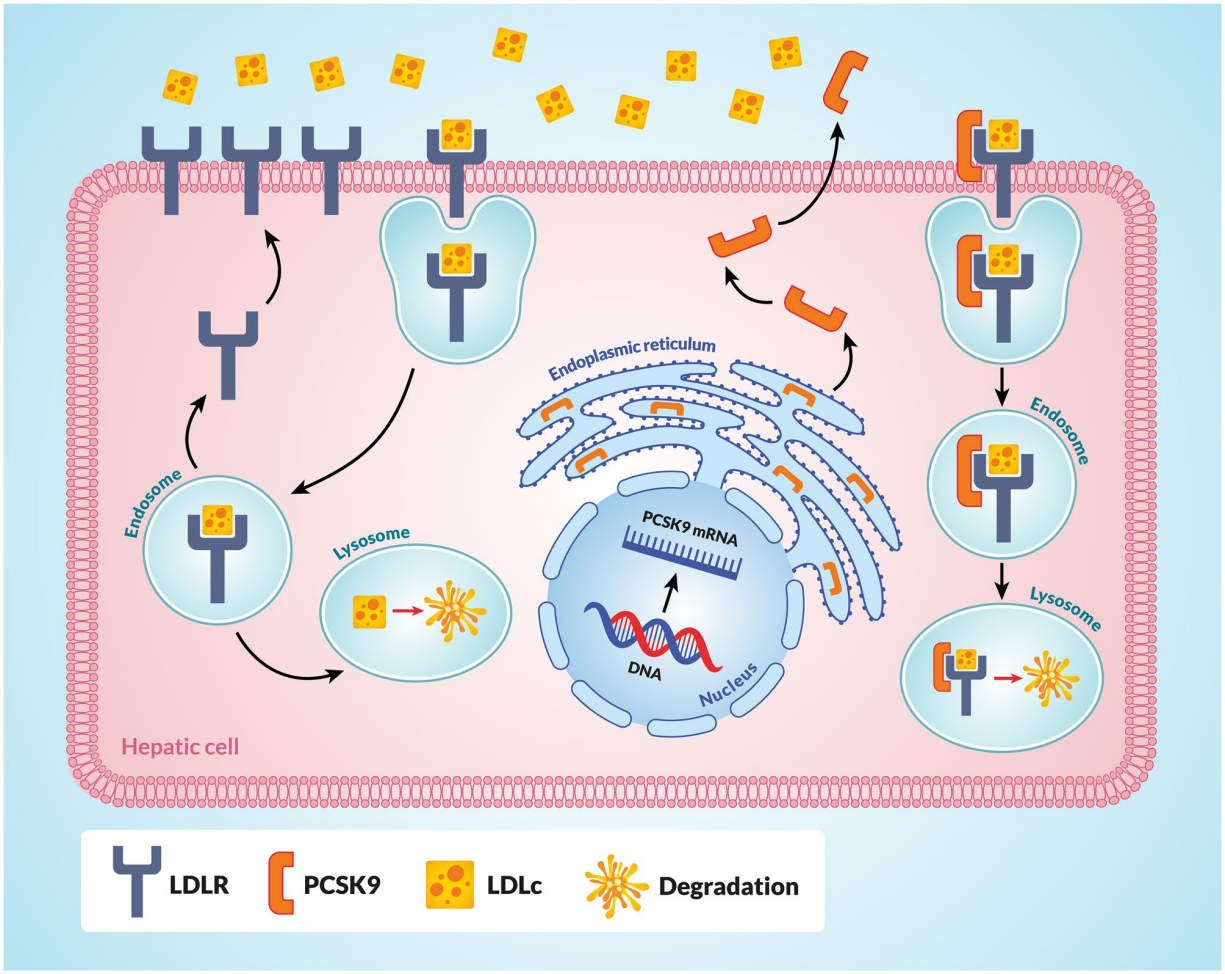
Mechanism of action of PCSK9 in a hepatocyte. PCSK9 is involved in regulating LDLc levels by binding to hepatic LDLR, which ultimately leads to its lysosomal degradation. When the LDLR-LDLc complex is formed, it is internalised and enters endosomes. There, in the acidic environment (pH 5.4), LDLR undergoes some conformational changes that lead to the release of LDLc from the complex. LDLc is then transported to lysosomes and undergoes degradation, while LDLR returns to the cell surface. When PCSK9 attaches to LDLR, this LDLR-PCSK9 complex also undergoes internalisation and enters endosomes, where the aforementioned acidic environment makes the binding between LDLR and PCSK9 much stronger. As a result, LDLR is unable to dissociate from PCSK9 and the entire LDLR-PCSK9 complex enters lysosomes and is degraded. As a result, LDLR is unable to return to the cell surface and bind to LDLc. Explanation of abbreviations: PCSK9, proprotein convertase subtilisin/keksin type-9; PCSK9 mRNA, mRNA for proprotein convertase subtilisin/keksin type-9; LDLc, low-density lipoprotein cholesterol; LDLR, LDL receptor

LDLR is a multi-domain protein, consisting of the extracellular domain (ECD), the epidermal growth factor (EGF) precursor domains, the O-linked sugar domain, the trans-membrane segment, and NPxY cytoplasmic sequences [1, 47]. The ECD contains the ligand binding domain involved in the interaction of LDLR with LDL and VLDL, whereas the EGF precursor domain consists of EGF-like repeats such as EGF-A and EGF-B and is involved in the interaction between LDR and PCSK9 [48]. Once formed, the LDLR-LDLc complex is internalised by clathrin-coated vesicles and enters endosomes. There, in an acidic environment (pH 5.4), LDLR undergoes some conformational changes that lead to the release of LDLc from the complex. LDLc is then transported to lysosomes and degraded, while LDLR returns to the cell surface [1] (Fig. 1). When PCSK9 binds to LDLR, this LDLR-PCSK9 complex also undergoes internalisation and enters endosomes, where the aforementioned acidic environment makes the binding between LDLR and PCSK9 much stronger. The binding between LDLR and PCSK9 initially occurs at pH 7.0, and then PCSK9 forms an anti-parallel beta-sheet and salt bridge with the EGF-A domain of LDLR in a calcium-dependent mechanism. In addition, the L108 residue in the pro-domain of PCSK9 binds directly to the L626 residue of LDLR. The acidic (pH 5.4) environment of the endosomes significantly enhances this coupling between PCSK9 and the ligand binding domain of LDLR. As a result, LDLR is unable to dissociate from PCSK9, and the entire LDLR-PCSK9 complex enters lysosomes and is degraded, so that LDLR is unable to return to the cell surface and bind to LDLc [49] (Fig. 1).

The LDLR family includes several trans-membrane receptors such as LDLR, VLDLR, LPR1-10, and sorLA/LR11, which participate in the endocytosis of various extracellular ligands. As in LDLR, the EGF-A domain is present in VLDLR, LRP1, and ApoER2 and has the ability to bind to PCSK9 [1]. LPR1 (as well as LPR2, LPR5, and LPR6) regulates mataloproteinases. LPR1 regulates extracellular levels of proteolytic enzymes including tissue-type plasminogen activator and matrix metalloproteinases-2, -9, and -13 (MMP-2, -9, and -13) [50]. LPR1 also interacts with other ligands such as lipoproteins and growth factors (transforming growth factor-beta1 [TGF-β1], platelet-derived growth factor [PDGF]) [51]. It is now believed that LPR1 has a protective effect on the vascular wall and prevents atherosclerosis [52]. The local expression of PCSK9 in macrophages within atheroma reduces LPR1 levels on the surface of mouse macrophages and affects the composition of atherosclerotic plaques [53]. Increased levels of LPR1 on the cell surface have been found in PCSK9 knockout mice [54]. However, the involvement of PCSK9 in LPR1 degradation has not been confirmed and requires studies in specific cells and tissues [1].

## The role of PCSK9 in cardiovascular diseases

### Heart failure

Clinical studies have recently shown that circulating PCSK9 levels are significantly higher in patients with heart failure (HF), mainly heart failure with reduced ejection fraction (HFrEF). Additionally, high PCSK9 levels are an adverse prognostic indicator in patients with HF exacerbation [55, 56]. However, there are also reports of LOF mutations that involve the disruption of local PCSK9 function in the heart and lead to lipid accumulation in cardiomyocytes [15]. In the absence of PCSK9, the expression of key receptors involved in lipid and lipoprotein uptake in cardiomyocytes increases, leading to lipid accumulation in the heart. In such a situation, cardiomyocytes switch from beta-oxidation of fatty acids to anaerobic glycolysis, which is unable to fully meet the energy requirements of the cell. This metabolic switch leads to certain morphological adaptations in the heart, including an increase in left ventricular wall thickness in order to maintain ejection fraction as is common in HFpEF [15]. This was confirmed in a study in which echocardiographic parameters of individuals with the PCSK9 LOS variant R46LW were assessed and compared with age- and sex-matched individuals without this mutation. Those with the R46LW variant had a significantly higher left ventricular mass index than the healthy subjects, whereas left ventricular ejection fraction values did not differ between the groups [15]. Studies in genetically induced PCSK9-deficient mice have shown that an inability to synthesise PCSK9 results in this metabolic switch in cardiomyocytes, as well as structural changes in the heart and impaired exercise tolerance compared to the wild-type mouse strain. This means that the lack of PCSK9 causes HFpEF [15]. Studies have also been conducted using an experimental model where the mice had a selective lack of PCSK9 expression only in the liver with otherwise preserved PCSK9 production in other tissues. When such an experimental model was used, there were no metabolic abnormalities in cardiomyocytes, changes in oxygen consumption, morphological changes in the heart, or impaired exercise tolerance compared to the wild-type strain mice [15]. This is an important observation because this model replicates the situation that occurs with anti-PCSK9 therapies currently used in clinical practice. These therapies reduce the concentration of circulating PCSK9 produced in the liver and do not interfere with local extrahepatic PCSK9 production. This allows us to exclude the adverse effect of the treatment-derived reduction in the level of circulating PCSK9 on the cardiac metabolism and is consistent with cardiac function data obtained in clinical trials evaluating anti-PCSK9 therapies.

In a similar experimental model, mice with genetically induced PCSK9 deficiency had histological abnormalities of the pancreatic islets and impaired insulin secretion [11]. There was also a poorer pancreatic islet beta-cell function in humans with the PCSK9 LOF variant R46L, when compared with individuals without this mutation. In an experimental model using mice with genetic variants characterised by the selective absence of PCSK9 expression only in the liver with preserved PCSK9 production in other tissues, no morphological changes or pancreatic islet dysfunction were observed [11]. This model replicates the situation that occurs with anti-PCSK9 therapies. These therapies reduce the concentration of circulating PCSK9 produced in the liver and do not interfere with local, extrahepatic PCSK9 production. Currently, available clinical trial data have not shown an increased incidence of diabetes in patients receiving anti-PCSK9 therapies [11]. However, a Mendelian randomisation study using data from different studies and genetic registries showed that certain LOF variants of PCSK9 (rs11583680, rs11591147, rs2479409, and rs11206510) are associated with higher fasting glucose concentration and body weight and with an increased risk of type 2 diabetes [12]. Other meta-analyses have also confirmed the link between the PSCK9 LOF variant rs11591147 and a higher risk of type 2 diabetes [13].

PCSK9 has been implicated in biological processes such as inflammation, apoptosis, pyroptosis, ferroptosis, and autophagy. Each of these processes is important in the development and progression of HF [55, 57–60].

**Fig. 2:**
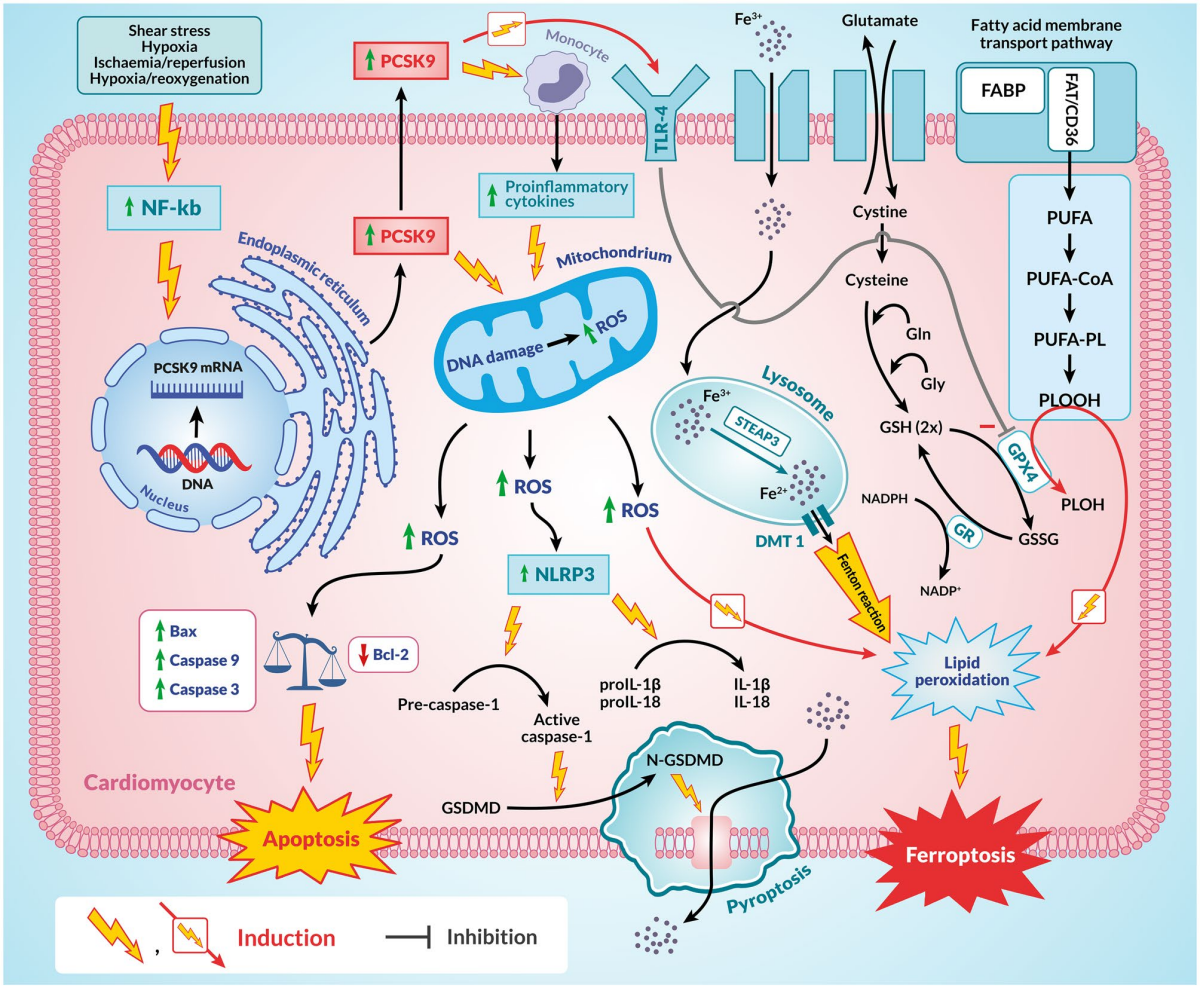
The role of PCSK9 in the development and progression of heart failure through its involvement in such biological processes as apoptosis, pyroptosis, and ferroptosis of cardiomyocytes. Particularly high levels of PCSK9 expression have been found in cardiomyocytes exposed to such harmful factors as hypoxia, ischaemia/reperfusion, and hypoxia/reoxygenation. An increased release of PCSK9 by cardiomyocytes promotes cardiomyocyte apoptosis by activating the NF-kB pathway and, by the stimulation of macrophages, releasing pro-inflammatory cytokines. PCSK9 increases mitochondrial ROS release and contributes to increased expression of the pro-apoptotic caspase 9, caspase 3, and Bax and decreased expression of the anti-apoptotic protein Bcl-2. PCSK9 may also be involved in the development and progression of HF by initiating mitochondrial DNA damage and releasing ROS, leading to NLRP3 activation and pyroptosis. Activation of pyroptosis in cardiomyocytes begins with accumulation of the NLRP3 inflammasome with the involvement of DAMPs, leading to activation of caspase-1. Activated caspase-1 causes the conversion of GSDMD to N-GSDMD, which leads to the formation of holes in the cell membrane. A damaged cell membrane allows the contents of the cell to escape, leading to a strong inflammatory response. The second effect of caspase-1 activation is the conversion of inactive forms of IL-1 β and IL-18 into their active forms. Together, this leads to cell death. PCSK9 also affects another process associated with the development and progression of HF-ferroptosis. This is a type of programmed cell death in which iron overload induces the Fenton reaction, ROS production, and lipid peroxidation, ultimately leading to cell death. The mechanisms regulating ferroptosis in cardiomyocytes are complex, involving several metabolic pathways and ROS production. The major source of ROS in the cell is the mitochondria. PCSK9 induces mitochondrial dysfunction and increases ROS production in cardiomyocytes. It is thought that PCSK9 may regulate ferroptosis in cardiomyocytes by regulating mitochondrial function and ROS production. PCSK9 may also regulate ferroptosis in cardiomyocytes via TLR4 which inhibits GPX4, thereby enhancing ferroptosis. Explanation of abbreviations: PCSK9, proprotein convertase subtilisin/keksin type-9; NF-kB, nuclear factor kappa B; ROS, reactive oxygen species; NLRP3, NOD-like receptor family, pyrin domain containing 3; DAMPs, damage-associated molecular patterns; GSDMD, Gasdermin D; N-GSDMD, N-terminal Gasdermin D; IL-1β, interleukin-1beta; IL-18, interleukin-18; STEAP3, the six-transmembrane epithelial antigen of prostate family member 3; TLR4, toll-like receptor 4; GPX4, glutathione peroxidase 4; NADP^+^, nicotinamide adenine dinucleotide (oxidised); NADPH, nicotinamide adenine dinucleotide (reduced); Glu, glutamate; Gly, glycine; GR, glutathione reductase; GSSG, glutathione disulfide; GSH, glutathione; FABP, fatty-acid-binding protein; FAT, ester of fatty acid; CD36, fatty acid translocase, PUFA, polyunsaturated fatty acid; PUFA-Pl, polyunsaturated fatty acid—containing phospholipids; PLOOH, phospholipid hydroperoxide; PLOH, phospholipid alcohol

Initially, PCSK9 was confirmed to be involved in the process of neuronal apoptosis and is therefore also known as neuronal apoptosis-regulated convertase-1 (NARC-1) [23]. The involvement of PCSK9 in apoptosis of other cell types, such as ECs and cardiomyocytes, was later confirmed. Particularly high expression of PCSK9 was found in cardiomyocytes exposed to damage by factors such as hypoxia, ischaemia/reperfusion, and hypoxia/reoxygenation [55, 57, 61]. In addition, high expression of PCSK9 in the border zone of myocardial infarction has been confirmed in animals and humans [58, 62]. This increased PCSK9 expression was associated with increased caspase-3 expression in the myocardium of patients with end-stage HF, indicating a link between PCSK9 and cardiomyocyte apoptosis [63–65] (Fig. 2). Furthermore, in an animal model of myocardial injury caused by ischaemia/reperfusion, the use of a PCSK9 inhibitor reduces the expression of apoptosis-related proteins such as Bax and caspase-3 and the myocardial infarct size [61]. In in vitro studies using co-culture of cardiomyocytes and macrophages, exposure of these cells to hypoxia/reoxygenation results in an increased release of PCSK9 by cardiomyocytes and can promote cardiomyocyte apoptosis by activating the nuclear factor kappa B (NF-kB) pathway and stimulating macrophages to release pro-inflammatory cytokines [57]. In cell culture, hydrogen sulphide (H_2_S) also significantly inhibits PCSK9 expression via the PI3K/AKT-SREBP-2 pathway [66]. In a mouse model of left ventricular injury by ischaemia/reperfusion, H_2_S significantly reduced infarct size and protected against deterioration of left ventricular systolic function [67]. This suggests that PCSK9 is involved in the process of apoptosis and that cardiomyocyte apoptosis can be regulated by inhibiting PCSK9 expression via the PI3K/AKT-SREBP-2 pathway.

Research with human umbilical vein endothelial cells (HUVECs) cell culture has confirmed that oxLDL induces endothelial cell apoptosis and increases PCSK9 and LOX-1 expression in a dose-dependent manner [68]. In these cells, oxLDL-mediated increased expression of the pro-apoptotic protein Bax, increased activity of caspase9 and caspase3, and decreased expression of the anti-apoptotic protein Bcl-2 were shown. However, blocking PCSK9 production by specific siRNAs inhibited oxLDL-induced apoptosis of ECs. This suggests, therefore, that PCSK9 promotes apoptosis in HUVECs via the Bcl/Bax-Caspase9-Caspase3 pathway [37, 68]. Apoptosis of ECs promotes endothelial dysfunction, which underlies the development of atherosclerotic lesions in arteries [37].

The participation of PCSK9 in pyroptosis, which is involved in the development and progression of HF, has also been confirmed [55]. Pyroptosis is a pro-inflammatory, lytic mode of programmed cell death initiated by activation of caspase 1. Characteristic of this process is the formation of cracks and pores in the cell membrane, resulting in the release of cell contents into the intercellular space. This ultimately leads to a strong inflammatory response and cell death [55, 69–71] (Fig. 2). The activation of pyroptosis in cardiomyocytes begins with the accumulation of NOD-like receptor family, pyrin domain containing 3 (NLRP3) inflammasome aided by damage-associated molecular patterns (DAMPs), which results in the activation of caspase-1. Activated caspase-1 leads to the conversion of Gasdermin D (GSDMD) to N-terminal GSDMD (N-GSDMD), which creates holes in the cell membrane. Through this damaged cell membrane, the contents of the cell escape, resulting in a strong inflammatory response. The second effect of caspase-1 activation is the conversion of inactive forms of IL-1β and interleukin-18 (IL-18) into their active forms (Fig. 2). Together, this leads to cell death. The importance of pyroptosis in the progression of HF has been demonstrated in several experimental models. A mouse model of induced myocardial hypertrophy confirmed a significant increase in the expression of activated caspase-1, IL-1β, and N-GSDMD in the myocardium, demonstrating the importance of pyroptosis in the development of HF based on myocardial hypertrophy. In addition, this experimental model showed that the inhibition of caspase-1 or NLRP3 reduced the severity of pyroptosis, resulting in less myocardial hypertrophy and less severe HF [72]. A significant activation of NLRP3 in the myocardium and increased expression of caspase-1, IL-1β, and IL-18 in patients with end-stage HF due to dilated cardiomyopathy indicates the role of pyroptosis in HF [73]. A mouse model of doxorubicin-induced dilated cardiomyopathy showed similar results where knockout of the gene for NLRP3 inhibited pyroptosis and reduced the extent of doxorubicin-induced left ventricular dysfunction [73]. PCSK9 is directly involved in the initiation of pyroptosis at the level of NLRP3 activation. In both animals and humans, there is a significant concomitant increase in the expression of PCSK9, NLRP3, caspase-1, IL-1β, and IL-18 in both chronic myocardial ischaemia and acute myocardial infarction [55]. In addition, exposure to exogenously administered hrPCSK9 increased reactive oxygen species (ROS) production during hypoxia, which was significantly reduced in PCSK9 gene knockout. This PCSK9 gene knockout inhibited pyroptosis under these experimental conditions and protected against deterioration of left ventricular function [74]. Thus, PCSK9 may be involved in the development and progression of HF by initiating mitochondrial DNA damage and ROS release, leading to NLRP3 activation and pyroptosis (Fig. 2).

The phenomenon of pyroptosis has also been reported in ECs [75–78]. Studies with HUVECs confirmed that not only does oxLDL significantly enhance the death of these cells, but also that inclisiran significantly inhibits oxLDL-induced HUVEC cell death and oxLDL-induced pyroptosis [75]. The pyroptosis of ECs results in the release of leukocyte chemotactic factors, adhesion molecules, and pro-inflammatory cytokines, which is critical for the initiation and progression of atherosclerotic lesions [76]. In HUVECs, inclisiran significantly inhibited pyroptosis-related proteins such as NLRP3, N-GSDMD, IL-1β, and IL-18 [75].

PCSK9 also affects ferroptosis, another process associated with the development and progression of HF. Ferroptosis is a type of programmed cell death in which iron overload of the cell induces the Fenton reaction, ROS production, and lipid peroxidation, ultimately leading to cell death (Fig. 2) [55, 79, 80]. In the course of ferroptosis, mitochondria undergo characteristic morphological changes, including damage to their outer membrane. A relationship between ferroptosis and neurodegenerative diseases, cancer, and cardiovascular diseases, including HF, has been reported [55, 80]. Several experimental animal models of HF show that cardiomyocyte ferroptosis is associated with HF progression [81, 82]. In mice, the use of the ferroptosis inhibitor ferrostatin-1 or the iron chelator dexrazoxane significantly reduces the severity of HF caused by ischaemia/reperfusion-induced left ventricular injury [83]. The mechanisms regulating ferroptosis are complex and involve various metabolic pathways and ROS production. The main source of ROS in the cell is the mitochondria, which are the main site of fatty acid metabolism and provide the specific lipid precursor for ferroptosis. In addition, mitochondria are rich in iron, which is also essential for ferroptosis. In mice, PCSK9 increases ROS production in cardiomyocytes, whereas PCSK9 inhibition reduces ROS production and increases cardiomyocyte survival [58]. In addition to cardiomyocytes, this increase in survival, under the influence of PCSK9 inhibition, has also been confirmed in ECs and VSMCs. In these cells, exposure to hrPCSK9 or PCSK9 overexpression results in a significant increase in ROS production and induction of mitochondrial dysfunction [36, 84]. It is thought that PCSK9 may regulate the process of ferroptosis in cardiomyocytes by regulating mitochondrial function and ROS production, thereby influencing the progression of HF. The suppression of toll-like receptor 4 (TLR4) by siRNA significantly inhibits ferroptosis in cardiomyocytes and reduces the severity of HF. TLR4 is regulated by PCSK9. The inhibition of PCSK9 results in decreased TLR4 expression in mouse aorta, whereas overexpression of PCSK9 results in upregulation of TLR4 in macrophages [60]. PCSK9 may influence the progression of HF through its involvement in TLR4-mediated regulation of cardiomyocyte ferroptosis, which suppresses glutathione peroxidase 4 (GPX4) and thus enhances ferroptosis (Fig. 2). The inhibition of PCSK9 may decrease TLR4 expression and restore the correct level of GPX4 activity, thus suppressing ferroptosis [55].

PCSK9 also regulates the process of autophagy in the heart, which is involved in the development of HF. The role of this process varies at different stages of HF [85, 86]. At the cellular level, autophagy is a process that allows the cell to survive adverse conditions by forming autophagy bodies through the endocytosis of damaged and unnecessary proteins or organelles. These bodies then fuse with lysosomes, and the lysosomal hydrolase system degrades their contents. At the initial stage of the damaging agent’s action on the cells, this process has a protective effect. However, under prolonged and intensive exposure to the damaging agent, the enhanced autophagy process contributes to worsening cellular and organ damage. In an ischemia/reperfusion-based model of left ventricular damage, moderate autophagy has a protective effect at the ischaemic stage, whereas over-activated autophagy at the later reperfusion stage exacerbates left ventricular damage and contributes to the development of HF [87]. There is a significant increase in the severity of autophagy in patients with left ventricular hypertrophy and HF in the decompensated phase. It is thought that the severity of autophagy may increase up to tenfold in patients with severe end-stage HF [86, 88]. PCSK9 is closely linked with the process of cardiomyocyte autophagy. With exposure to damaging factors such as ischaemia, reperfusion, inflammation, ROS, or shear stress, the expression of PCSK9 in cardiomyocytes increases significantly [36, 57, 61, 89]. In patients with acute myocardial infarction, there is a significant increase in PCSK9 expression and increased autophagy in the border zone of the myocardial infarction. It is possible that PCSK9 may induce and enhance autophagy through the ROS-ATM-LKB1-AMPK signalling axis and thus influence the extent of infarct damage and deterioration of cardiac function [58]. In addition, disruption of LDLR recycling to the surface of cardiomyocytes by PCSK9, thereby interfering with cholesterol uptake by cardiomyocytes, may contribute to the upregulation of autophagy and thus to the progression of HF [55]. The involvement of PCSK9 in the development and progression of HF is also suggested by its association with dynamin-related protein 1 (DRP-1), which is a regulator of PCSK9 secretion. In an animal model of doxorubicin-induced HF, DRP-1 was associated with mitochondrial autophagy. Knockdown of the DRP-1 gene attenuates the doxorubicin-induced autophagy process and protects cardiomyocytes from mitochondrial autophagy-induced death [90]. Similarly, in a model of left ventricular hypertrophy and HF, both PCSK9 expression and the intensity of mitochondrial autophagy induced by oxidative stress and DRP-1 were shown to be high in the hearts of the mice studied. The use of evolocumab, a PCSK9 inhibitor, reduced DRP-1 phosphorylation and the severity of mitochondrial autophagy, suggesting that PCSK9 and DRP-1 are involved in the regulation of mitochondrial autophagy in cardiomyocytes and in the development and progression of HF [91]. However, the detailed mechanisms by which PCSK9 is involved in the regulation of autophagy and the development of HF are not fully understood and require further study.

## Atherosclerosis

**Fig. 3:**
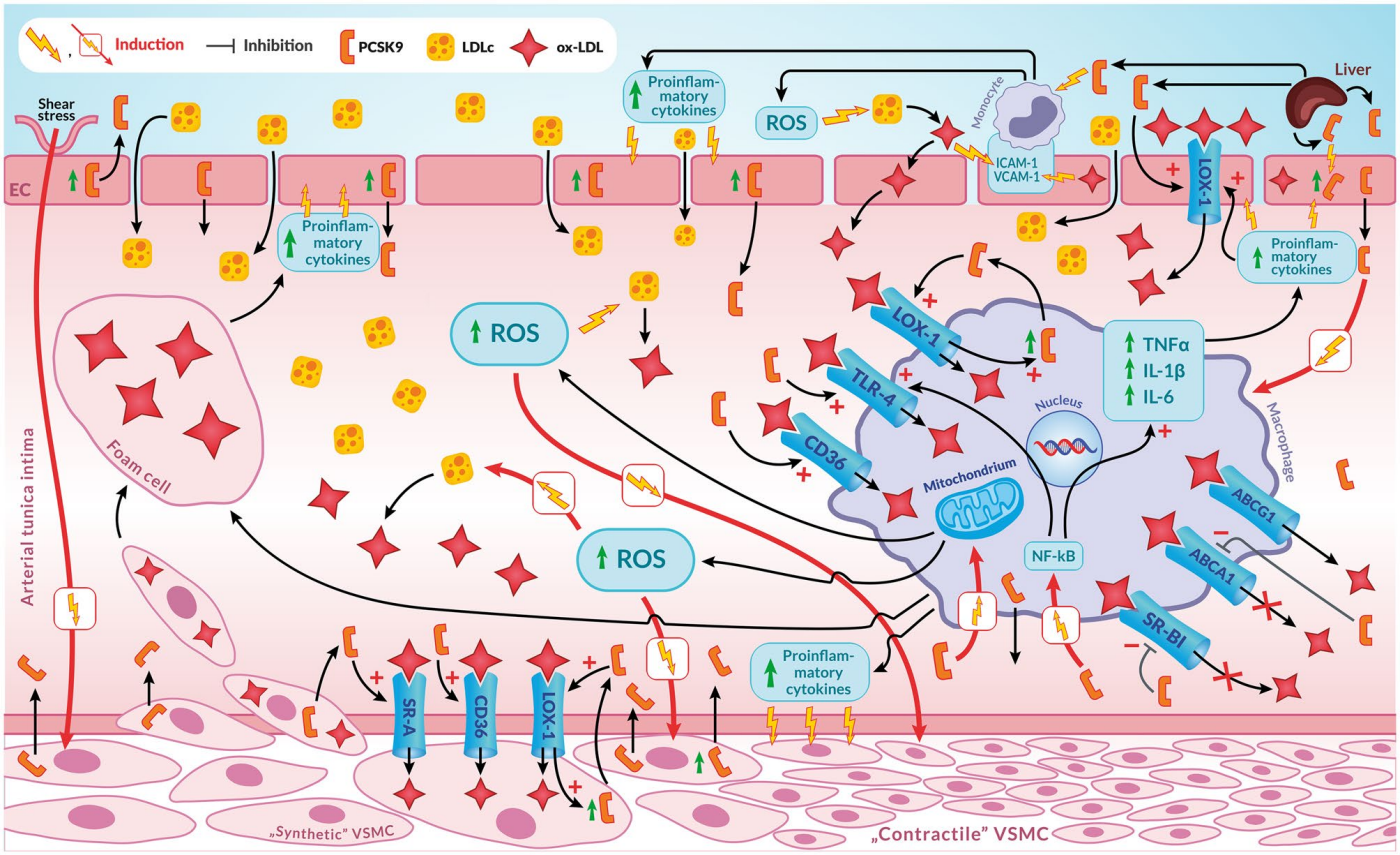
Mechanisms of the atherosclerotic and pro-inflammatory effects of PCSK9 on the arterial wall. VSMCs are the major source of PCSK9 in the vascular wall, while PCSK9 expression has also been confirmed in endothelial cells and macrophages. In the early stages of atherosclerosis, a key process is the formation of foam cells in the vascular wall. These foam cells are mainly created by macrophages engulfing oxLDL. OxLDL enters not only macrophages but also monocytes, VSMCs and fibroblasts via SRs such as LOX-1, SR-A, CD36, and TLR4. LOX-1 is the most important of these SRs. PCSK9 increases the expression of all SRs, but especially LOX-1, in macrophages and VSMCs. In these cells, PCSK9 and LOX-1 increase each other’s expression, resulting in increased uptake of oxLDL by these cells. This interaction between PCSK9 and LOX-1 has important implications for the development of atherosclerotic lesions. LOX-1 is the major oxLDL receptor in ECs, but is also present at high levels in VSMCs. LOX-1 activation enhances oxLDL uptake, increases adhesion molecule expression, mitochondrial ROS production, and inflammation. Increased inflammation, in turn, increases LOX-1 expression, creating a self-perpetuating atherosclerotic ‘vicious cycle’. PCSK9, interacting with LOX-1, is directly involved in this process. PCSK9 also promotes foam cell formation by another mechanism. PCSK9 inhibits cholesterol efflux in macrophages by inhibiting the expression of ABCA1, one of the membrane transporters through which most cholesterol efflux in macrophages occurs. PCSK9 also inhibits, to a lesser extent, the expression of SR-BI, another membrane transporter through which cholesterol efflux occurs. This promotes the formation of foam cells. PCSK9 increases the secretion of pro-inflammatory cytokines by macrophages. At the cellular level, this stimulating effect of PCSK9 on pro-inflammatory cytokine production is mediated by NF-kB. PCSK9 induces NF-kB translocation to the nucleus in macrophages, resulting in increased mRNA levels for pro-inflammatory cytokines and TLR4. The resulting inflammation is also associated with increased production of ROS in the mitochondria. ROS induce endothelial dysfunction, induce and sustain inflammation, increase inflammatory cell infiltration and activation, and enhance apoptosis of ECs and VSMCs. Increased levels of ROS also increase the expression of PCSK9 and LOX-1. Pro-inflammatory cytokines, ROS, and also shear stress induce the differentiation of contractile VSMCs into synthetic VSMCs, which synthesise extracellular matrix, proteases, and cytokines, and have a greater capacity for proliferation and migration. Synthetic VSMCs also have enhanced lipid synthesis and increased expression of SRs, which contribute to foam cell formation. PCSK9, which is significantly upregulated in VSMCs under shear stress, induces the differentiation of VSMCs from a contractile phenotype to synthetic VSMCs and enhances VSMC proliferation and migration. Explanation of abbreviations: PCSK9, proprotein convertase subtilisin/keksin type-9; LDLc, low-density lipoprotein cholesterol; oxLDL, oxidised LDL; SRs, scavenger receptors; SR-A, class A scavenger receptor; SR-BI, class B scavenger receptor type I; LOX-1, lectin-like oxLDL receptor-1; ECs, endothelial cells; VSMCs, vascular smooth muscle cells; ROS, reactive oxygen species; ABCA1, ATP-binding cassette A1; ABCG1, ATP-binding cassette sub-family G member 1; NF-kB, nuclear factor kappa B; TLR4, toll-like receptor 4; ICAM-1, intercellular adhesion molecule 1; VCAM-1, vascular cell adhesion molecule 1; TNFa, tumor necrosis factor alpha; IL-1β, interleukin-1beta; IL-6, interleukin-6

Initially, the involvement of PCSK9 in the initiation and progression of atherosclerotic lesions in the arteries was exclusively linked to the role of PCSK9 in enhancing hepatic LDLR degradation and thereby increasing plasma levels of LDLc, a recognised atherogenic factor. Atherosclerosis is a chronic disease of the arteries that begins with endothelial lesions associated with the accumulation, oxidation, and glycation of LDLc in the inner membrane of the arteries. Endothelial damage promotes the accumulation of LDLc in the ECs as well as in the arterial tunica intima under them, leading to spontaneous oxidation and oxLDL formation [21, 92, 93]. Such modified LDL particles are then recognised by various receptors, such as CD36, TLR4, and LOX-1, which stimulate the production of pro-inflammatory cytokines and the internalisation of oxidative species by vascular wall cells and phagocytic cells. This is accompanied by increased expression of adhesion molecules and secretion of chemotactic factors for monocytes. The monocytes recruited to the inner membrane are transformed into macrophages, which engulf oxLDL and become foam cells (Fig. 3). This ultimately leads to the death of these cells and the release of pro-inflammatory cytokines that perpetuate the inflammatory process and contribute to the recruitment of more monocytes to the site of injury and the formation of more foam cells. The accumulation of LDLc in the arterial intima and the inflammation sustained by pro-inflammatory cytokines also induce the migration of VSMCs and their phenotypic changes, leading to the production of pro-inflammatory cytokines, the uptake of oxLDL, and the formation of foam cells. All this further exacerbates the damage to the arterial wall [94, 95] (Fig. 3). Lipid deposits in the arterial wall develop into atherosclerotic plaques, which may cause an acute ischaemic episode (e.g. myocardial infarction or ischaemic stroke) [3, 21].

It was initially believed that the role of PCSK9 in atherosclerosis was only linked to its involvement in LDLc metabolism [3]. There is now, however, increasing evidence that PCSK9 also has direct, atherosclerotic effects on the arteries, and this may be partially independent of its hyperlipidaemic effect. This suggests that PCSK9 is directly involved in the formation of atherosclerotic plaques in arteries at each stage of the process [3]. In the vessel wall, VSMCs are the main source of PCSK9, and PCSK9 expression has also been confirmed in ECs and macrophages, highlighting the importance of PCSK9 in the initiation and progression of the atherosclerotic process [36, 96–98].

As mentioned above, a key process in the early stages of atherosclerosis is the formation of foam cells in the vessel wall, which mainly form when macrophages engulf oxLDL. The oxLDL enters not only macrophages but also monocytes, VSMCs, and fibroblasts via SRs such as SR-A, scavenger receptor class B type I (SR-BI), CD68, CD36, and LOX-1. Of all these receptors, LOX-1 is the most important in the uptake of oxLDL by macrophages. Its activity is also associated with increased production of adhesion molecules and oxidative stress. PCSK9 increases the expression of all SRs, but, most significantly, LOX-1 in monocytes and VSMCs. In these cells, PCSK9 and LOX-1 enhance each other’s expression, leading to increased uptake of oxLDL by these cells [3, 99] (Fig. 3). In VSMCs, lipopolysaccharide (LPS) significantly increases the expression of both PCSK9 and LOX-1, whereas inhibition of PCSK9 by siRNA reduces LOX-1 expression in these cells approximately fivefold [99]. The exposure of VSMCs to hPCSK9 additionally causes a significant increase in LOX-1 and vascular cell adhesion molecule 1 (VCAM-1) expression in these cells, over and above that induced by their previous exposure to LPS [99]. This interaction between PCSK9 and LOX-1 has important implications for the development of atherosclerotic lesions. LOX-1 is the major oxLDL receptor in ECs, but is also present in large amounts in VSMCs, which becomes particularly important when the endothelium is damaged, as in the early stages of atherosclerotic lesion formation. In both these cells, LOX-1 activation enhances oxLDL uptake, increases adhesion molecule expression, ROS production, and inflammation [99–101]. Increased inflammation in turn increases LOX-1 expression, creating a self-perpetuating atherosclerotic ‘vicious circle’. PCSK9 is directly involved in this process by interacting with LOX-1. Knockdown of PCSK9 almost completely blocks oxLDL uptake and VCAM-1 expression, whereas increased PCSK9 expression significantly increases oxLDL uptake and VCAM-1 expression [99].

PCSK9 also promotes foam cell formation by another mechanism. PCSK9 inhibits cholesterol efflux in macrophages by inhibiting ATP-binding cassette A1 (ABCA1), one of the membrane transporters through which most cholesterol efflux in macrophages occurs [3]. This efflux is mainly mediated by membrane transporters such as ABCA1, ATP-binding cassette G1 (ABCG1), and SR-BI. PCSK9 inhibits cholesterol efflux in macrophages by inhibiting ABCA1 expression and, to a lesser extent, SR-BI expression [3, 102] (Fig. 3). This promotes foam cell formation.

PCSK9 also has the ability to downregulate ApoER2 in a mechanism similar to that by which it reduces LDLRs on the cell surface. ApoE binding to ApoER2 reduces lipid accumulation in macrophages, inhibits foam cell formation, and changes the phenotype of macrophages from pro-inflammatory (M1) to anti-inflammatory (M2) [103, 104]. This confers protective, anti-atherosclerotic effects. PCSK9 inhibits these anti-atherosclerotic effects of ApoE by eliminating the availability of ApoER2.

## Vascular inflammation

PCSK9 has the ability to induce inflammation in the vessel wall in the early stages of the development of atherosclerotic lesions, by a mechanism independent of its hyperlipidaemic effect [3]. The increased uptake of oxLDL by monocytes/macrophages described above stimulates ECs to produce pro-inflammatory cytokines. PCSK9 can also directly increase the expression of pro-inflammatory cytokines in monocytes. Of particular importance are the so-called inflammatory monocytes, Ly6C(hi), which accumulate at the site of inflammation at an early stage of atherosclerotic lesion formation. By secreting specific chemotactic factors, they recruit other populations of monocytes to these locations, which then infiltrate the site of atherosclerotic damage and become macrophages that secrete large amounts of pro-inflammatory cytokines [105, 106] (Fig. 3). PCSK9 increases the secretion of pro-inflammatory cytokines by macrophages in response to LPS. In ECs and VSMCs, LPS also significantly increases PCSK9 expression [36, 99]. PCSK9 increases the secretion of pro-inflammatory cytokines such as TNF-α, IL-1β, and IL-6 by macrophages, while decreasing the secretion of anti-inflammatory cytokines such as interleukin-10 (IL-10) [37, 107]. These pro-inflammatory actions of PCSK9 are cholesterol-independent [53, 107]. At the cellular level, this effect of PCSK9 in stimulating pro-inflammatory cytokine production is mediated by NF-kB, which controls the transcription of several genes for pro-inflammatory cytokines, acute phase proteins, adhesion molecules, regulators of apoptosis, and cell proliferation [108]. PCSK9 induces NF-kB translocation to the nucleus in macrophages, resulting in increased mRNA levels for pro-inflammatory cytokines and TLR4 [21, 109] (Fig. 3). NF-kB inhibition has been shown to reduce LPS-, oxLDL-, and TNF-α-induced PCSK9 expression. This highlights the importance of TLR4/NF-kB pathway activation in the pro-inflammatory and atherosclerotic effects of PCSK9 [3, 21, 109]. Inflammation increases VCAM-1 expression. PCSK9 also directly increases VCAM-1 expression in VSMCs, which contributes to increased adhesion of lymphocytes, monocytes, and eosinophils to the endothelial surface [3]. Inflammation is also associated with increased production of ROS in the mitochondria [3]. ROS induce endothelial dysfunction, induce and maintain inflammation, increase inflammatory cell infiltration and activation, and enhance apoptosis of ECs and VSMCs. Increased ROS also augment the expression of PCSK9 and LOX-1 [3, 36]. Pro-inflammatory cytokines, ROS as well as shear stress induce the differentiation of contractile VSMCs into synthetic VSMCs, which synthesise extracellular matrix, proteases, and cytokines and have a greater capacity for proliferation and migration [110, 111] (Fig. 3). Synthetic VSMCs also have enhanced lipid synthesis and increased expression of SRs, which contribute to foam cell formation. PCSK9, whose expression in VSMCs is markedly increased under low shear stress, induces the differentiation of VSMCs from a contractile phenotype to synthetic VSMCs and enhances VSMC proliferation and migration [36, 112, 113].

## Coronary artery disease

Several aspects of PCSK9 have been considered in relation to CAD. Firstly, genetic studies have confirmed that certain PCSK9 variants (e.g. rs11206510) are associated with an increased risk of CAD [114]. In addition, certain PCSK9 GOF gene mutations (e.g. E670G) and subsequent higher plasma PCSK9 levels are linked to CAD severity [115–117].

Furthermore, a study using intracoronary ultrasound—the Atherosclerosis-Intravascular Ultrasound Study (ATHEROMO-IVUS) showed that high plasma PCSK9 concentrations positively correlate with necrotic core fraction and volume, and this correlation is independent of statin use and plasma LDLc concentration [118]. In another animal study, a PCSK9 inhibitor significantly and dose-dependently reduced the extent of atherosclerotic arterial damage and improved plaque morphology [119].

A study involving patients with acute coronary syndrome, PCSK9-REACT (Reactivity in Patients with Acute Coronary Syndrome Treated with Prasugrel and Ticagrelol), confirmed that higher plasma PCSK9 levels are associated with greater platelet reactivity and a higher risk of thrombotic events from atherosclerotic plaque within 1 year of an acute coronary syndrome event [120].

In a model of surgically induced myocardial infarction in rats and mice, there was a significant increase in plasma PCSK9 levels during the acute phase of myocardial infarction, with a peak concentration at 48 h after infarction [121]. The autophagy phenomenon, which is normally an important process regulating tissue rejuvenation, is also significantly upregulated in the course of myocardial infarction. The use of a PCSK9 inhibitor in mice reduces the area of myocardial infarction and decreases the excessive activation of the autophagy process [1]. This is supported by other studies in which a PCSK9 inhibitor reduced infarct size and improved cardiac function in rats with induced ischaemia/reperfusion injury [61].

## Cerebral artery disease

In cerebrovascular disease, single nucleotide polymorphisms (SNPs) in the PCSK9 gene are associated with stroke risk. To date, selected SNPs have been studied in specific populations. Some SNPs, such as the PCSK9 E670G SNP, are specifically associated with strokes caused by atherosclerotic lesions in large cerebral arteries and are not associated with strokes caused by atherosclerotic lesions in small cerebral arteries, as confirmed in a Belgian population [122]. In contrast, the PCSK9 ra1711503 and rs2479408 SNPs are associated with a higher risk of ischaemic stroke in a Chinese population [123]. Some PCSK9 SNPs, such as rs505151, positively correlate with both a higher risk of stroke and severe CAD in the Tunisian population [116]. It should be pointed out that the large variation in results relates to differences in age and ethnicity.

## Abdominal aortic aneurysm

Factors, which play an important role in the development of abdominal aortic aneurysms, include atherosclerotic lesions, upregulated MMP9, and apoptosis of VSMCs. These three factors are all affected by PCSK9, and therefore, PCSK9 may play a role in the formation of abdominal aortic aneurysms. Genetic studies have also identified 10 loci associated with a higher risk of abdominal aortic aneurysm [124]. Among the proteins to which PCSK9 binds, the importance of sortilin has been highlighted. Sortilin facilitates the translocation of PCSK9 from the endoplasmic reticulum to the cytoplasm, thereby enhancing the degradation of LDLR by PCSK9 and contributing to the more rapid development of atherosclerotic lesions. Also of importance is LRP1, which interacts with certain extracellular matrix enzymes such as MMP9. PCSK9 directly promotes apoptosis of VSMCs, which is critical for the development of abdominal aortic aneurysms. The effects of PCSK9 on VSMCs are complex and not fully understood. PCSK9 expression has been identified mainly in dedifferentiated VSMCs, but it is not known how PCSK9 induces the dedifferentiation process [36, 125]. Such dedifferentiated VSMCs lack contractility and have impaired extracellular matrix enzyme production, thus contributing to abdominal aortic aneurysm formation. Activation of the NF-kB pathway, which can be activated by PCSK9, is thought to be critical for this dedifferentiation process, as confirmed in macrophages [60, 126, 127]. Activation of the NF-kB pathway inhibits the expression of the gene for myocardin, which is an essential regulator of VSMC differentiation [126]. Myocardin may be involved in the PCSK9-induced dedifferentiation of VSMCs and thus contributes to the formation of abdominal aortic aneurysms [1].

## PCSK9 inhibition strategies

**Fig. 4:**
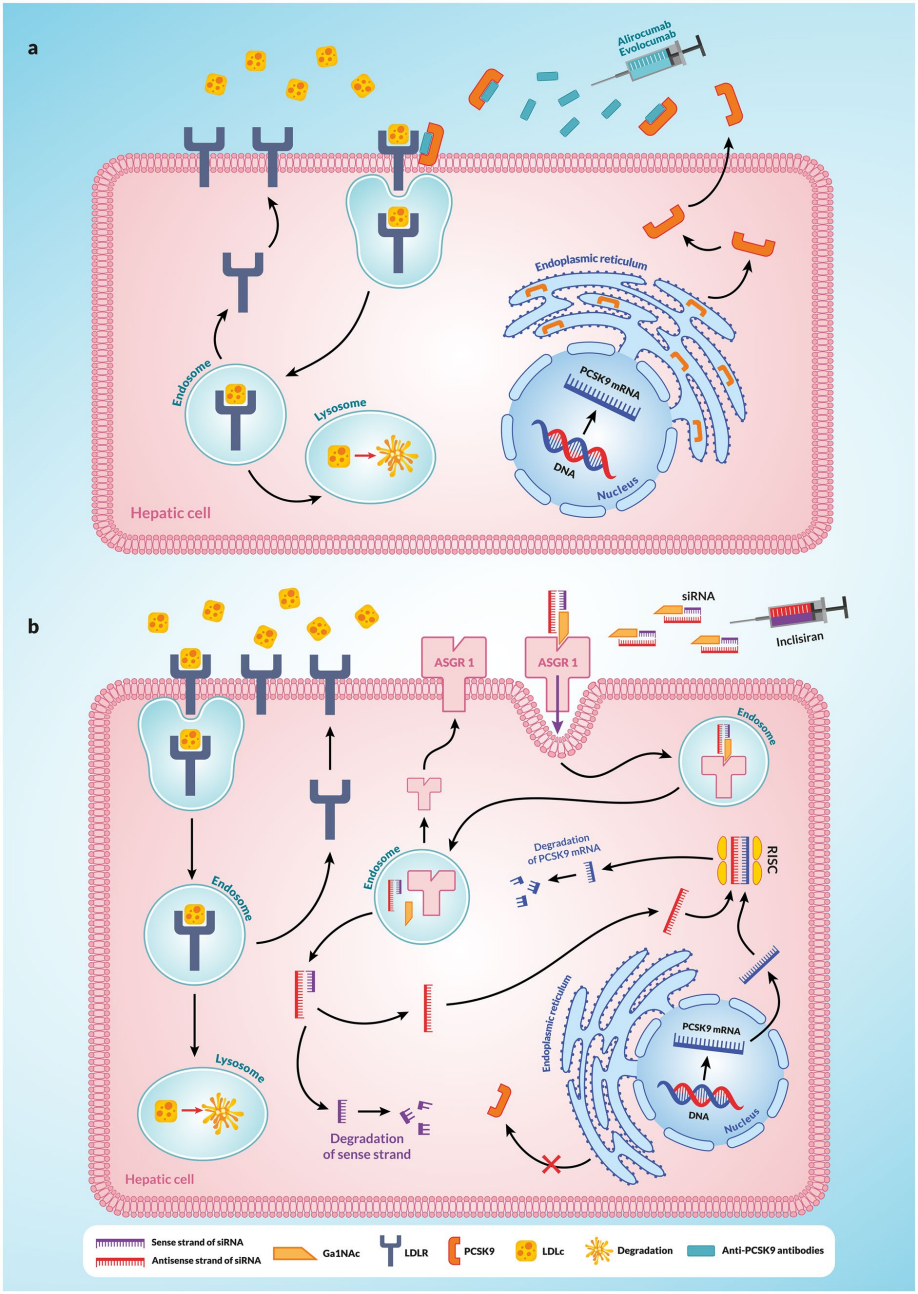
PCSK9 inhibition strategies. **a** The first strategy involves the use of anti-PCSK9 antibodies (evolocumab and alirocumab), which inhibit the interaction between circulating PCSK9 and LDLR on the surface of hepatocytes. As a consequence of these antibodies binding to PCSK9, they prevent PCSK9 from binding to LDLR on the surface of hepatocytes. This results in LDLR binding to LDLc and the formation of an LDLR-LDLc complex without PCSK9. This complex is then internalised and enters endosomes. There, in an acidic environment (pH 5.4), LDLR undergoes some conformational changes that lead to the release of LDLc from this complex. LDLc is then transported to lysosomes and undergoes degradation while LDLR, unhindered, returns to the cell surface. **b** The second strategy involves the administration of siRNA in the form of lipid nanoparticles (inclisiran). Inclisiran siRNA consists of complementary 21 sense and 23 antisense oligonucleotide sequences. To facilitate the uptake of siRNA by hepatocytes, Ga1NAc is fused to a sense strand and, because of this, it binds to ASGR1 which is highly expressed on hepatocytes. This allows specific delivery of siRNA to the liver. When endosomal uptake of siRNA occurs, small portions of siRNA are released into the cytoplasm where the dissociated antisense strand binds to PCSK9 mRNA and, with the participation of several proteins, forms RISC. This leads to degradation of PCSK9 mRNA resulting in the discontinuation of PCSK9 production. Explanation of abbreviations: PCSK9, proprotein convertase subtilisin/keksin type-9; LDLc, low-density lipoprotein cholesterol; LDLR, LDL receptor; PCSK9-mRNA, mRNA for proprotein convertase subtilisin/keksin type-9; siRNA, small interfering RNA; ASGR1, asialoglycoprotein receptor 1; Ga1NAc, N-acetylgalactosamine; RISC, RNA-induced silencing complex

Since the earliest and best-studied action of PCSK9 is its ability to bind to the hepatic LDLR and thereby increase plasma concentrations of atherogenic LDLc, strategies to eliminate PCSK9 have long been an attractive approach to treating hypercholesterolaemia. Two different strategies have been successfully implemented, each providing potent, long-term, and safe inhibition of PCSK9. The first strategy involves the use of anti-PCSK9 antibodies (evolocumab and alirocumab) that inhibit the interaction between circulating PCSK9 and LDLR on the surface of hepatocytes (Fig. 4a). The second strategy involves the administration of siRNAs in the form of lipid nanoparticles (inclisiran) (Fig. 4b). The siRNAs contained in inclisiran consist of complementary 21 sense and 23 antisense oligonucleotide sequences, which are designed for long persistence and low immunogenicity. To facilitate the uptake of siRNA by hepatocytes, the sense strand is fused to triantennary N-acetylgalactosamine (Ga1NAc) during production. Ga1NAc binds to the asialoglycoprotein receptor 1 (ASGR1), which is highly expressed on hepatocytes [2, 128, 129]. This allows specific delivery of siRNA to the liver. Following endosomal uptake of siRNAs, small portions of siRNAs are released into the cytoplasm where the dissociated antisense strand binds to PCSK9 mRNA and forms the RNA-induced silencing complex (RISC) with the participation of several proteins. This leads to PCSK9 mRNA degradation that is prolonged over time [130] (Fig. 4b). Inclisiran can therefore be administered by subcutaneous injection twice a year. Both strategies reduce plasma LDLc concentrations by 50–60% beyond the reduction achieved with statins alone [2].

There are other new PCSK9 inhibition strategies, and these are described below in the section ‘Future Perspectives’.

## Clinical trials

Two humanised anti-PCSK antibodies (evolocumab and alirocumab) are currently in clinical use, and their efficacy and safety have been confirmed in clinical trials in patients with and without familial hypercholesterolaemia (FH) [2, 16–18]. All these clinical trials have confirmed the exceptional efficacy of these drugs in reducing LDLc concentrations by 50–60%. The excellent tolerability of these drugs, administered as subcutaneous injections every 2 or 4 weeks, has also been confirmed. This high efficacy and very good tolerability of the treatment were observed both in patients receiving one of these drugs as monotherapy and in patients receiving one of them in combination with a statin or with a statin and ezetimibe. The efficacy of evolucumab and alirocumab in reducing LDLc was even greater when these drugs were used in combination with a statin. This is interesting because statin treatment is known to activate the SREBP-2 pathway and thereby increase PCSK9 expression [2, 131, 132].

In the FOURIER study of 27,564 statin-treated patients (receiving at least 20 mg of atorvastatin), the patients were randomised to receive evolocumab subcutaneously at a lower dose of 140 mg once every 2 weeks or at a higher dose of 420 mg once a month, or a placebo [2]. In this study, evolocumab significantly reduced LDLc to mean levels of 30 mg/dl after 48 months of follow-up. There was also a significant reduction in the risk of cardiovascular events in patients with known atherosclerotic cardiovascular disease and in patients with type 2 diabetes [2, 16, 17]. The relative risk of this primary endpoint was reduced by 15%. The secondary composite endpoint in this study was cardiovascular death, myocardial infarction, or stroke. A 20% relative risk reduction in this secondary endpoint was achieved. The number needed to treat (NNT) was 5–7 [2]. This study also confirmed that patients at higher cardiovascular risk or with more advanced atherosclerotic cardiovascular disease had greater clinical benefit from evolocumab [2]. However, a relative increase in cardiovascular mortality in the evolocumab-treated group was found, although this increase was statistically non-significant (RR 1.20, 95% CI 0.95 to 1.51, *p* = 0.13) [133].

The ODYSSEY trial, which studied 18,924 patients with a history of acute coronary syndrome in the 1–12 months prior to randomisation, evaluated alirocumab administered subcutaneously at a dose of 75 mg every 2 weeks compared with a placebo [2, 18]. Patients participating in this study used atorvastatin 40 mg or rosuvastatin 20 mg, and unlike in the FOURIER trial, the statin dose could be adjusted during the study. At 48 months, LDLc was reduced to a mean of 66 mg/dl. In this study, the relative risk of the primary composite endpoint, which consisted of death from acute coronary syndrome, myocardial infarction, ischaemic stroke, or unstable angina requiring hospitalisation, was reduced by 15% with the alirocumab treatment. In addition, the alirocumab treatment produced a 16.2% reduction in absolute risk of death in patients with concomitant CAD, peripheral artery atherosclerosis, and cerebral artery atherosclerosis [134].

An evaluation of the efficacy and safety of inclisiran was conducted as part of the ORION clinical trial programme [19]. This programme consists of Phase II and Phase III clinical trials, some of which have been completed and some of which are ongoing. The first of these, ORION-1, evaluated the use of inclisiran as a single dose and as two doses 90 days apart in high-risk cardiovascular patients with elevated LDLc levels. A 50% reduction in LDLc levels was achieved and maintained for at least 6 months after two doses of inclisiran. The median duration of maintaining an LDLc reduction of at least 39 mg/dl from baseline was 6–9 months for a single dose and 5–10 months for two doses, indicating the long-term effect of inclisiran [19, 20].

The efficacy and safety of inclisiran compared to placebo in patients with heterozygous FH and atherosclerotic cardiovascular disease who were receiving maximally tolerated hypolipemic therapy was also evaluated (ORION-9, -10, -11). In the study group, inclisiran resulted in a 47.9% reduction in LDLc assessed at 510 days of follow-up in patients who received 30 mg of inclisiran on days 1, 90, 270, and 450 of the study [135]. This effect was independent of the type of genetic defect underlying FH. This study will be continued in the ongoing ORION-8 study, in which patients from the ORION-9, -10, and -11 studies will receive long-term treatment with 30 mg of inclisiran twice a year until day 990. This study is designed to evaluate the efficacy and safety of long-term treatment with inclisiran in the study population [19]. The ongoing ORION-4 study is also designed to evaluate whether long-term use (median treatment duration of 5 years) of inclisiran is safe and reduces the risk of major cardiovascular events in patients with known atherosclerotic cardiovascular disease. The study is expected to be completed in December 2024 [19]. The VICTORION-2P study is a phase III clinical trial to assess the clinical benefit of inclisiran in patients with confirmed atherosclerotic cardiovascular disease who tolerate hypolipemic therapy (high-dose statins with or without ezetimibe) well and for whom inclisiran is an adjunct therapy. The planned follow-up for this study is 72 months [19]. Two clinical trials are also planned to evaluate the efficacy and safety of inclisiran in adolescents, aged 12–17 years with homozygous FH (ORION-13) and heterozygous FH (ORION-16) [19].

As can be seen, many clinical trials evaluating inclisiran are still ongoing. However, the results of the completed trials have provided the basis for the approval of inclisiran by the Food and Drug Administration (FDA) and the European Medicines Agency (EMA) for the treatment of adult patients with primary hypercholesterolaemia (including heterozygous FH) or mixed hyperlipidaemia who do not achieve their treatment goal on the maximum tolerated dose of a statin or who cannot use a statin due to intolerance or contraindications [19].

There was also a randomised, prospective, single-centre, open-label clinical trial that did not look at the effect of PCSK9 inhibitors on lipid parameters or typical cardiovascular endpoints. This was the EXpanded Combination of Evolocumab plus Empagliflozin on Diabetes Trial (EXCEED-BHS3), which evaluated, in a group of 110 patients with type 2 diabetes, whether the addition of evolocumab to empagliflozin improved endothelial function, as assessed by flow-mediated dilation (FMD), compared with the use of empagliflozin alone [136]. Follow-up in this study was only 16 weeks. The combination of evolocumab and empagliflozin was shown to improve endothelial function as assessed by FMD compared to empagliflozin alone [136]. Due to the small size of the study population and the short duration of the study, a long-term clinical trial with combined assessment of FMD and hard endpoints is needed.

## Adverse effects of PCSK9 inhibitors

The available data on the adverse effects of PCSK9 inhibitors come mainly from clinical trials evaluating the efficacy and safety of this class of drugs. In the FOURIER and ODYSSEY trials, which evaluated evolocumab and alirocumab, respectively, the incidence of adverse events such as the worsening of diabetes in patients with diabetes or new-onset diabetes in patients without diabetes as well as of hepatic disorders, neurocognitive disorders, haemorrhagic strokes, cataracts, allergic reactions, or any adverse event leading to withdrawal from the study was not significantly different between the active treatment and placebo groups [16–18]. Likewise, there were no significant differences in laboratory abnormalities between the two groups [16, 18]. Only local injection site reactions were more common in patients treated with both evolocumab and alirocumab compared to a placebo (evolocumab 2.1% vs. 1.6%, *p* < 0.001; alirocumab 3.8% vs. 2.1%, *p* < 0.001) [16, 18]. These local injection site reactions usually consisted of itching, swelling, or redness and were mild in severity. They also often spontaneously resolved themselves [16, 18]. In the FOURIER study, there were no differences in body weight changes between the evolocumab and placebo groups [17]. In this study, however, as mentioned above, the authors found a statistically non-significant relative increase in cardiovascular mortality in the evolocumab-treated group [133]. A pre-specified analysis from ODYSSEY OUTCOME was also performed, taking into account the stratification of patients after acute coronary syndrome according to concomitant atherosclerosis in another vascular bed (monovascular disease, polyvascular disease in 2 beds, polyvascular disease in 3 beds) [134]. This analysis showed no difference in the incidence of the above-mentioned adverse events between the alirocumab and placebo groups, with the exception of local injection site reactions. These reactions were more common in the alirocumab group regardless of the presence and extent of concomitant atherosclerosis in another vascular bed [134].

The adverse event data for inclisiran are from the ORION clinical trial programme [19, 20, 135]. In these studies, the incidence of adverse events assessed using system-organ class was similar in the inclisiran and placebo groups. There were also no significant differences in laboratory abnormalities between the two groups [135]. Again, only the incidence of local injection site reactions was significantly higher in the inclisiran group compared to the placebo group. In ORION-9, the incidence was 17.0% in the inclisiran group versus 1.7% in the placebo group. The severity of these local injection site reactions was rated mild to moderate, with no cases of severe or persistent reactions [135].

## Future perspectives

In addition to the anti-PCSK9 antibodies (evolocumab, alirocumab) or siRNA (inclisiran), there are attempts to use other methods to disable PCSK9 activity. These include attempts to develop an anti-PCSK9 vaccine that could eliminate PCSK9 activity in the long term [2]. To date, promising results have been obtained in mice and macaques, by using, among other things, bacteriophage virus-like particles that display peptides derived from PCSK9. Studies to develop a vaccine against PCSK9 are also using modified lipid nanoparticles and effective capsid-like particles [137–139].

An oral PCSK9 inhibitor, MK-0616, has also been evaluated in a randomised Phase 2b study [140]. MK-0616 is an orally bioavailable macrocyclic peptide with the ability to bind to PCSK9. Previous studies evaluating the administration of a single dose of this inhibitor achieved a reduction in free PCSK9 levels of more than 90% [140]. In the randomised Phase 2b study, MK-0616 demonstrated statistically significant reductions in serum LDLc concentrations compared to placebo at all doses tested, as assessed at week 8 of treatment. The resulting reductions in serum LDLc concentrations were 41.2%, 55.7%, 59.1%, and 60.9% for MK-0616 doses of 6 mg, 12 mg, 18 mg, and 30 mg, respectively (*p* < 0.001 for each group compared) [140]. This oral PCSK9 inhibitor was well tolerated during 8 weeks of treatment and an additional 8 weeks of follow-up [140].

Attempts have also been made to use a cholesteryl ester transfer protein (CETP) inhibitor. A new CETP inhibitor, K-312, reduces PCSK9 expression in hepatocytes by decreasing SREBP-1 and SREBP-2 activity [141]. In animal studies, K-32 significantly reduced serum LDLc levels and inhibited the progression of atherosclerosis [141].

In addition, berberine, an isoquinoline plant alkaloid isolated from Huanglian (Coptis chinesis), exerts some pharmacological effects on lipid and carbohydrate metabolism [141]. Oral use of berberine in patients with hypercholesterolaemia results in a reduction in total cholesterol, triglycerides, and LDLc. Berberine reduces levels of both hepatocyte nuclear factor- alpha (HNF-α) and SREBP-2, resulting in the potent inhibition of PCSK9 transcavitation. In vitro studies have confirmed that berberine dose-dependently reduces PCSK9 mRNA levels [141].

Adnectin, a synthetic protein based on the 10th type III domain of human fibronectin, is also currently under investigation [141]. Adnectin BMS-962476 is a PCSK9-targeting polypeptide that binds to PCSK9 with high affinity. It interferes with the interaction between extracellular PCSK9 and the EGF-a domain of LDLR, thereby preventing PCSK9-induced LDLR degradation [141]. In the first clinical trial in which adnectin BMS-962476 was administered either subcutaneously or intravenously, free PCSK9 levels were reduced by more than 90%. No significant side effects of the drug were observed in this study [141]. These initial observations need to be confirmed in a longer follow-up. However, preliminary results suggest that adnectin BMS-962476 may be a promising new drug which could be an alternative to anti-PCSK9 antibodies in achieving significant reductions in serum LDLc concentrations.

In addition, attempts have been made to use the clustered regularly interspaced short palindromic repeats (CRISPR)-associated system (Cas) of gene editing [2, 142]. There are reports of the use of adeno-associated viral vector-9 to deliver CRISP-Cas9 into the liver of mice [142]. In utero injections of an adenoviral vector expressing the SpCas9 base editor 3, which allows single-base conversions without double-strand breaks, have also been used [143]. In both cases, a significant reduction in PCSK9 expression and LDLc levels was achieved in the mice tested. It was also confirmed that intravenous administration of non-viral lipid nanoparticles (LNPs) carrying Cas9/sgRNA ribonucleoprotein complexes significantly reduced plasma PCSK9 concentrations in mice [144]. The importance of this method is highlighted mainly because, despite the LNPs not being delivered directly to the liver, good results have been obtained. Recently, a single intravenous administration of LNPs containing the more advanced CRISPR-ABE8.8 system was also used in cynomolgus monkeys. This intravenous administration of the aforementioned system successfully modified the gene for PCSK9 and resulted in a 90% reduction in plasma PCSK9 levels and a 60% reduction in plasma LDLc levels sustained for more than 8 months [145]. It appears that this efficacy in reducing plasma LDLc levels could be even greater if a way can be developed to deliver these LNPs more specifically to the liver [2].

These initial encouraging reports need to be confirmed in further studies. If the usefulness and applicability of these methods in humans is confirmed, they are likely to be a powerful tool not only for treating hypercholesterolaemia, but also for eliminating other additional adverse cardiovascular effects of PCSK9.

## Conclusions

Since the first observations confirming the association of PCSK9 with enhanced hepatic LDLR degradation and elevated plasma LDLc concentrations, a number of additional actions of PCSK9 have been identified that are central to the development of cardiovascular diseases. Many of these additional actions of PCSK9 are independent of its effect on LDLR. Of particular importance are the direct atherosclerotic and pro-inflammatory actions of PCSK9 in the cardiovascular system, as described above. For this reason, the possibility of using treatment methods aimed at eliminating PCSK9 in clinical practice is of particular importance. Current PCSK9-eliminating strategies based on anti-PCSK9 antibodies (evolocumab, alirocumab) or the use of siRNA (inclisiran) have been evaluated in numerous clinical trials and have had their efficacy and high safety profile confirmed. The efficacy of these strategies has been confirmed both in terms of reducing plasma LDLc concentrations and in terms of reducing cardiovascular risk. Given the above mechanisms of cardiovascular action of PCSK9, some of which are independent of its effects on LDLR and plasma LDLc concentrations, the strategies currently used to eliminate PCSK9 may have broader significance beyond the treatment of hypercholesterolaemia. This protective effect on the cardiovascular system may even be enhanced if new PCSK9 elimination strategies currently being tested in animals can be introduced into clinical practice.

Further research is needed to better understand the mechanisms of PCSK9 action in the cardiovascular system and to develop new clinically feasible strategies to eliminate PCSK9. This may allow further improvement in the prognosis of patients with HF and with other cardiovascular diseases.

## Data Availability

Not applicable.
